# Prävalenz kognitiver Beeinträchtigungen in der pflegerischen Akutversorgung – Analyse und Vergleich von Routinedaten

**DOI:** 10.1007/s00391-020-01722-5

**Published:** 2020-04-04

**Authors:** Tobias Mai, Christa Flerchinger

**Affiliations:** Stabsstelle Pflegeentwicklung, Pflegedirektion des Universitätsklinikums der Goethe-Universität Frankfurt, Theodor-Stern-Kai 7, 60590 Frankfurt am Main, Deutschland

**Keywords:** Kognitive Störung, Screeninginstrument, Mini-Mental-Status-Test, Pflegeprozess, Krankenhaus, Cognitive disorder, Screening instrument, Mini mental state examination, Nursing process, Hospital

## Abstract

**Hintergrund:**

Aufgrund des demografischen Wandels ist von einer zunehmenden Prävalenz stationärer Patienten mit kognitiven Beeinträchtigungen auszugehen. Für eine bestmögliche Versorgung gilt es, diese Patientengruppe mit einem routinemäßigen Verfahren frühzeitig zu erkennen.

**Methode:**

Die aktuelle Studie untersuchte die Prävalenzrate von kognitiven Beeinträchtigungen bei stationären Patienten >65 Jahre, die mit dem Mini-Mental-Status-Test (MMST) während der pflegerischen Aufnahme untersucht wurden. Anhand der Screeningquote wurde die Bereitschaft von Pflegekräften, den MMST als Routineinstrument zu verwenden, überprüft. Die Quote wurde zudem mit der Anzahl der kodierten F‑Diagnosen der ICD-10-GM verglichen. Diese retrospektive Studie wurde vom Oktober 2018 bis März 2019 an einer Universitätsklinik durchgeführt.

**Ergebnisse:**

Von 7311 stationären Patienten >65 Jahre wurden 11,7 % gescreent. Die Prävalenz kognitiver Beeinträchtigungen betrug 20,7 % und war höher als die Prävalenzrate von Demenz und Delir aufgrund medizinischer Diagnosen (*p* < 0,001). Mit 11,7 % ist die Bereitschaft des Pflegepersonals, den MMST zur Einschätzung kognitiver Beeinträchtigungen während der Patientenaufnahmen einzusetzen, gering.

**Diskussion:**

Die höhere Prävalenz bestätigt die Notwendigkeit, Patienten mit kognitiven Störungen zu erkennen. Die geringe Bereitschaft, den MMST anzuwenden, legt jedoch nahe, dass zum einen ein kürzeres Instrument verwendet und zum anderen Pflegenden mehr Informationen zu kognitiv beeinträchtigten Patienten vermittelt werden sollten.

## Abstract (en)

**Background:**

Due to demographic changes an increasing prevalence of hospitalized patients suffering from cognitive impairments must be assumed. To enable the best possible treatment a routine procedure is necessary to detect this group of patients early on admission.

**Method:**

The current study assessed the prevalence rate of cognitive impairment in inpatients >65 years who were tested by the mini mental state examination (MMSE) during admission. Based on the screening quota the willingness of nurses to use the MMSE as a routine instrument was tested. The quota was also compared to the number of diagnoses coded as F in the ICD-10-GM. This retrospective study was conducted from October 2018 to March 2019 at a university hospital.

**Results:**

Of the 7311 inpatients >65 years, 11.7% were screened. The prevalence of cognitive impairments was 20.7% and was higher than the prevalence rate of dementia and delirium on the basis of medical diagnoses (*p* <0.001). With only 11.7% the willingness of the nursing staff to use the MMSE to assess cognitive impairments during patient admission was low.

**Conclusion:**

The higher prevalence confirms the necessity to detect patients with cognitive disorders; however, the low willingness to use the MMSE suggests that a shorter instrument should be used and more information should be provided to nurses about patients with cognitive impairments.

## Hintergrund

Laut Statistischem Bundesamt ist rund die Hälfte aller stationären Patienten über 65 Jahre [21]. Mit steigendem Alter geht ein erhöhtes Risiko für Komorbiditäten einher. Studien belegen, dass bis zu 40 % der Patienten über 65 Jahre in Krankenhäusern von kognitiven Störungen wie Demenz oder Delir betroffen sind [3, 4, 17, 18, 20]. Um ihren besonderen Bedürfnissen gerecht zu werden und ein Höchstmaß an Patientensicherheit zu gewährleisten, gilt es, diese Patienten frühzeitig zu erkennen. Ein routinemäßiges Screening auf kognitive Beeinträchtigungen erleichtert eine passgenaue Versorgungs- und Pflegeplanung. Die Prävalenzrate demenziell betroffener Patienten über 65 Jahre im Akutkrankenhaus liegt mit 3,5 % für deutsche Universitätskliniken deutlich unter den in der internationalen Literatur beschriebenen Raten [23]. Dies lässt vermuten, dass Patienten mit kognitiven Störungen teils unerkannt und möglicherweise unterversorgt bleiben [7, 10]. Doch gerade Patienten mit kognitiven Beeinträchtigungen wie Demenz erfordern eine höhere Aufmerksamkeit, da sie ein höheres Risiko für Delir, Stürze, freiheitsentziehenden Maßnahmen, längere Verweildauern und höhere Sterblichkeitsraten haben [14, 18]. Aus diesem Grund ist ein systematisches Screening dieser Hochrisikogruppe erforderlich, um Hinweise auf eine kognitive Beeinträchtigung frühzeitig zu erkennen [15].

### Erkennen als Aufgabe der professionellen Pflege

Die Einschätzung von Ressourcen, Bedürfnissen und Versorgungserfordernissen der Patienten ist eine explizite Aufgabe der professionellen Pflege. Pflegende sind verpflichtet, anhand möglichst objektiver Parameter den psychosozialen und körperlichen Zustand von Patienten zu beurteilen, um die weitere Versorgung zu planen. Im Rahmen dieser Pflegeprozessverantwortung muss die Pflegeplanung alle Pflegeanforderungen aufzeigen und nicht nur die Maßnahmen, die zur Unterstützung der medizinischen Verfahren erforderlich sind oder im Kontext der Einweisungsdiagnose stehen. Aus diesem Grund wurde an einer Universitätsklinik auf Basis eines Pilotprojektes ein Prozess beschrieben, der Pflegende verpflichtet, am Tag der stationären Aufnahme eines älteren Menschen über 65 Jahre ein kognitives Screening durchführen. Entsprechend der Empfehlung der medizinischen S3-Leitlinie „Demenz“ war es nicht Ziel, eine Demenzerkrankung zu diagnostizieren [2].

## Ziele und Fragestellungen

Ziel dieser Studie ist es zum einen, die Notwendigkeit eines Screenings durch die Prävalenz kognitiver Beeinträchtigungen aufzuzeigen, indem der Mini-Mental-Status-Test (MMST) als Instrument zur Identifizierung betroffener Patienten in einem Akutkrankenhaus verwendet wird. Um zu untersuchen, ob diese Prävalenzrate vergleichbar ist mit jener, die sich aus den nach der International Classification of Diseases and Related Health Problems (ICD) in der 10. Version und den German-Modification-Kodierungen für Demenz und Delir ergibt (F-Diagnosen nach ICD-10-GM), werden diese beiden Raten miteinander verglichen. Es soll geprüft werden, ob sich die Verteilung der kognitiven Beeinträchtigungen in der MMST-Gruppe nicht von der Verteilung der F‑Diagnosen in der Gruppe der über 65-Jährigen unterscheidet. Zum anderen wird die Bereitschaft des Pflegepersonals, den MMST regelhaft anzuwenden, anhand der Screeningquote in verschiedenen Organisationseinheiten betrachtet.

Im Rahmen dieser Evaluation stellen sich folgende Fragen:

1. Wie hoch ist die Prävalenzrate von Patienten über 65 Jahre mit kognitiven Beeinträchtigungen, wenn der MMST als routinemäßiges Screeninginstrument im Pflegeprozess in der Akutversorgung eingesetzt wird?
2. Welche Aussage ist aus der Screeningquote über die Bereitschaft der Pflegenden, den MMST als Routinescreeninginstrument einzusetzen, abzuleiten?
3. Gibt es einen Unterschied zwischen der Prävalenzrate von kognitiv beeinträchtigten Patienten mittels der Einschätzung durch den MMST und der Prävalenzrate von medizinischen Diagnosen von Demenz oder Delir?

## Methoden

### Auswahl des Instruments

Um eine Doppeldokumentation zu vermeiden, wurde sich für eines der 3 im ICD-10-GM-Code-Handbuch des Deutschen Instituts für Medizinische Dokumentation und Information (DIMDI) vorgeschriebenen Screeninginstrumente entschieden [6]. Der ICD-Code U51 „Kognitive Beeinträchtigung“ des ICD-10-GM kann kodiert werden, wenn die kognitive Beeinträchtigung entweder mit dem erweiterten Barthel-Index (EBI), dem kognitiven Functional Independence Measure (FIM) oder mittels MMST nachgewiesen wird [6].

In einem Pilotprojekt wurden die Praktikabilität und Verwendbarkeit des MMST und des EBI durch Pflegekräfte eingeschätzt. Hierfür wurden Pflegende auf 4 Stationen (Kardiologie und Traumatologie) hinsichtlich der Durchführung von MMST und EBI geschult. Sie wendeten jeweils 2 Wochen den MMST und 2 Wochen den EBI im Rahmen der Patientenaufnahme an. Die anschließenden Gruppendiskussionen mit Pflegenden führten zur Entscheidung, den MMST als objektiveres Routineinstrument für das kognitive Screening in den Pflegeprozess auf den somatischen Stationen einzubinden. Jeder Patient 65 Jahre und älter sollte bei Aufnahme von einer Pflegeperson mit dem MMST gescreent werden. Für die Implementierung wurden Multiplikatoren aus mehreren Bereichen in 2‑stündigen Kursen in der Durchführung des MMST geschult.

### Mini-Mental-Status-Test

Der MMST wurde zunächst 1975 von Folstein et al. für Forschungszwecke entwickelt [9]. Der MMST erfasst in einem Interview mit dem betroffenen Menschen mit 30 Items 5 Dimensionen: Orientierungsfähigkeit, Merkfähigkeit, Aufmerksamkeit und Rechenfähigkeit, Erinnerungsfähigkeit sowie Verstehen und Handlungsfähigkeit. In Summe sind maximal 30 Punkte zu erreichen. Je niedriger die Punktezahl, desto eher ist die Wahrscheinlichkeit für kognitive Auffälligkeiten resp. Demenz. Der MMST zeigt die beste Sensitivität und Spezifität bei einem Grenzwert <24 Punkten (0,92; 0,96) [1]. Die „Interrater“-Reliabilität des MMST ist hoch (r = 0,83) [9]. Studien zeigen, dass der MMST von Pflegenden nach Schulung durchgeführt werden kann [13]. Darüber hinaus ist der MMST bei Ärzten bekannter als der EBI oder der FIM und erleichtert den interprofessionellen Austausch über den Versorgungsplan der Patienten.

### Datenerhebung

Die Studie basiert auf einem retrospektiven Beobachtungsdesign und nutzt die Daten aus der elektronischen Patientenakte. Die Datenerhebung umfasst den Zeitraum von 6 Monaten von Oktober 2018 bis März 2019. Eingeschlossen wurden alle Patientenakten von Patienten 65 Jahre und älter, die in diesem Zeitraum in einer Universitätsklinik in Deutschland auf somatischen Stationen behandelt und bereits entlassen wurden (*n* = 7311). Die aus den Patientenakten zusammengestellten Datensätze umfassten Angaben zu Alter, Geschlecht, Station und Organisationsbereich, Vorliegen des MMST, Punktwert des MMST, Vorliegen einer ICD-10-GM-Demenz- oder ICD-10-GM-Delirdiagnose (Tab. 1). Um evtl. eine relevantere Altersgrenze für ein obligates Screening zu erkennen, wurde das Alter in 6 jeweils enge Altersbereiche gruppiert: 65 bis 69 Jahre, 70 bis 74 Jahre, 75 bis 79 Jahre, 80 bis 84 Jahre, 85 bis 89 Jahre und 90 Jahre und älter.

**Table 1:** 

ICD-Code	ICD-Text
F00	Demenz bei Alzheimer-Krankheit
F00.0	Demenz bei Alzheimer-Krankheit, früher Beginn
F00.1	Demenz bei Alzheimer-Krankheit, später Beginn
F00.2	Demenz bei Alzheimer-Krankheit, atypisch
F00.9	Demenz bei Alzheimer-Krankheit, nicht näher bezeichnet
F01	Vaskuläre Demenz
F01.0	Vaskuläre Demenz, akuter Beginn
F01.1	Multiinfarktdemenz
F01.2	Subkortikale vaskuläre Demenz
F01.3	Gemischte (kortikale und subkortikale) vaskuläre Demenz
F01.8	Sonstige vaskuläre Demenz
F01.9	Vaskuläre Demenz, nicht näher bezeichnet
F02	Demenz bei sonstigen andernorts klassifizierten Krankheiten
F02.0	Demenz bei Pick-Krankheit
F02.1	Demenz bei Creutzfeldt-Jakob-Krankheit
F02.2	Demenz bei Huntington-Krankheit
F02.3	Demenz bei Parkinson-Krankheit
F02.4	Demenz bei Krankheit durch das Humane-Immundefizienz-Virus (HIV)
F02.8	Demenz bei anderenorts klassifizierten Krankheitsbildern
F03	Nicht näher bezeichnete Demenz
F05	Delir, nicht durch Alkohol oder sonstige psychotrope Substanzen bedingt
F05.0	Delir ohne Demenz
F05.1	Delir bei Demenz
F05.8	Sonstige Formen des Delirs
F05.9	Delir, nicht näher bezeichnet

*ICD* International Classification of Diseases and Related Health Problems, *GM* German Modification

Da eine Sekundärdatenanalyse von der Richtigkeit der Dokumentation in der Patientenakte und der Kodierung der medizinischen Diagnosen abhängt, ist sie fehleranfällig. Diesem Aspekt wurde durch die Prüfung der Plausibilität und der Vollständigkeit der MMST-Dokumente in den Patientenakten entsprochen. Unvollständige Dokumente führten zum Ausschluss des Falles (*n* = 45). Einige MMST konnten laut Dokumentation aufgrund motorischer Beeinträchtigung nicht abgeschlossen werden. Einige wurden aus nichtbeschriebenen Gründen abgebrochen. Darüber hinaus wird bei der Interpretation der Ergebnisse berücksichtigt, dass die Daten zu einem anderen Zweck als zu Forschungszwecken erhoben wurden. Alle Daten wurden vom Pflegepersonal in der Routineversorgung unabhängig vom Erkenntnisinteresse der Forschenden erfasst.

### Datenanalyse

Für die Analyse wurden die Datensätze pseudonymisiert. Die Auswertungen zur Prävalenz erfolgten deskriptiv. Die Grundgesamtheit sind alle stationären Fälle 65 Jahre und älter der somatischen Stationen, unabhängig von der Hauptdiagnose oder der Aufenthaltsdauer (*n* = 7311). Für die Analyse der Bereitschaft zu screenen wurden Organisationseinheiten zu medizinischen Fachgebieten zusammengefasst und die erfolgten MMST-Screenings im Verhältnis zu der Anzahl der Fälle 65 Jahre und älter dieser Bereiche gesetzt.

Die Gruppierung der MMST-Scores folgt den abrechnungsrelevanten Vorgaben des DIMDI und allgemeinen Regelwerken [1, 6], auch wenn insbesondere im Kontext von Stadieneinteilungen von Demenzerkrankungen 4‑stufige Differenzierungen empfohlen sind (keine, leichte, mittlere, schwere Beeinträchtigung) [16].

- 30 bis 24 Punkte: keine oder leichte kognitive Beeinträchtigung,
- 23 bis 17 Punkte: mittlere kognitive Beeinträchtigung,
- 16 bis 0 Punkte: schwere kognitive Beeinträchtigung.

Für die Betrachtung der Prävalenzraten kognitiver Beeinträchtigungen wurden die Verteilung der Häufigkeiten in der Gruppe der MMST-Screenings <24 Punkte mit den Häufigkeiten der kodierten Demenz- und Delirdiagnosen in allen Fällen der Grundgesamtheit verglichen. Um die Unterschiede zwischen diesen Verteilungen zu untersuchen, wurde ein Chi^2^-Anpassungstest (χ^2^) zum α‑Niveau = 0,05 angewendet („Goodness-of-fit“-Statistik). Die Referenzwerte für die erwartete Verteilung errechnen sich hierbei aus der Verteilung der F‑Diagnosen in der Grundgesamtheit. Ergänzend werden die Anteile der Fälle mit auffälligem MMST in der Gruppe der F‑Diagnosen betrachtet. Als Assoziationsmaß wird der Phi-Koeffizient (ɸ) für dichotome Merkmale berechnet. Zur besseren Interpretation von ɸ wird ɸ_max_ genutzt, welcher eine Aussage zu einem maximal möglichen Zusammenhang zulässt. Die Signifikanzprüfung erfolgt über die χ^2^-Verteilung zum α‑Niveau = 0,05.

Die Daten wurden in Microsoft Excel 2016 verwaltet und analysiert.

### Einhaltung ethischer Richtlinien

Das Hauptziel dieses Projektes war, mittels Optimierung der pflegerischen Statuserhebung im Rahmen des Pflegeprozesses für eine passgenauere Pflegeplanung eine Aussage über die Häufigkeit von kognitiven Beeinträchtigungen zu treffen. Das Vorgehen ist keine Interventionsstudie. Die Richtlinien des Datenschutzes wurden eingehalten. Es liegen keine ethischen Interessenkonflikte vor.

## Ergebnisse

### Prävalenzraten

Für den Untersuchungszeitraum wurden 7311 Akten stationärer Patienten über 65 Jahre in die Analyse eingeschlossen (Tab. 2). Das Durchschnittsalter betrug 75,8 Jahre (± 7,1). Nach Ausschluss unvollständiger Tests wurden 854 Fälle mit MMST in die Analyse einbezogen (11,7 %).

**Table 2:** 

	Alle Fälle		Fälle mit		MMST Fälle		Fälle mit		Fälle mit		Fälle mit	
	>65 Jahre		MMST^a^		<24 Punkte		F‑Diagnosen		Demenzdiagnose		Delirdiagnose	
*Anzahl*	*N* = 7311		*n* = 854		n = 177		*n* = 554		*n* = 311		*n* = 243	
*Rate (n/N) in %*	–		11,7 %		–		*7,6* *%*		4,3 %		3,3 %	
*Rate (n/n) in %*	–	–	–	–	*20,7* *%*		–	–	–	–	–	–
*Geschlecht*												
Männlich	4143	(56,7 %)	528	(61,8 %)	94	(53,1 %)	306	(55,2 %)	130	(41,8 %)	176	(72,4 %)
Weiblich	3167	(43,3 %)	326	(38,2 %)	83	(46,9 %)	247	(44,6 %)	181	(58,2 %)	66	(27,2 %)
Divers	1	(0,01 %)	–	–	–	–	1	(0,2 %)	–	–	1	(0,4 %)
*Alter (in Jahren)*												
Mean (±)	75,8	(7,1)	76,2	(6,9)	79,1	(7,6)	81,1	(8)	83,4	(7,5)	78,1	(7,7)
Min	65	–	65	–	65	–	65	–	65	–	65	–
Max	101	–	95	–	95	–	99	–	99	–	98	–
*Altersgruppen*												
65–69 Jahre	1722	(23,6 %)	182	(21,3 %)	20	(11,3 %)	58	(10,5 %)	14	(4,5 %)	44	(14,1 %)
70–74 Jahre	1647	(22,5 %)	173	(20,3 %)	36	(20,3 %)	66	(11,9 %)	29	(9,3 %)	37	(11,9 %)
75–79 Jahre	1774	(24,3 %)	230	(26,9 %)	36	(20,3 %)	124	(22,4 %)	55	(17,7 %)	69	(22,2 %)
80–84 Jahre	1241	(17,0 %)	155	(18,1 %)	36	(20,3 %)	125	(22,6 %)	78	(25,1 %)	47	(15,1 %)
85–89 Jahre	618	(8,5 %)	82	(9,6 %)	34	(19,2 %)	97	(17,5 %)	70	(22,5 %)	27	(8,7 %)
90 Jahre und älter	309	(4,2 %)	32	(3,7 %)	15	(8,5 %)	84	(15,2 %)	65	(20,9 %)	19	(6,1 %)
*Fachgebiete*												
Gefäßchirurgie	185	(2,5 %)	128	(15,0 %)	33	(18,6 %)	19	(3,4 %)	5	(1,6 %)	14	(5,8 %)
Urologie	284	(3,9 %)	148	(17,3 %)	14	(7,9 %)	12	(2,2 %)	11	(3,5 %)	1	(0,4 %)
Nuklearmedizin	128	(1,8 %)	51	(6,0 %)	4	(2,3 %)	0	(0,0 %)	0	(0,0 %)	0	(0,0 %)
Traumatologie	405	(5,5 %)	153	(17,9 %)	52	(29,4 %)	97	(17,5 %)	49	(15,8 %)	48	(19,8 %)
Kardiologie	893	(12,2 %)	133	(15,6 %)	23	(13,0 %)	47	(8,5 %)	26	(8,4 %)	21	(8,6 %)
Radiologie	257	(3,5 %)	34	(4,0 %)	2	(1,1 %)	0	(0,0 %)	0	(0,0 %)	0	(0,0 %)
Thorax‑/Herzchirurgie	416	(5,7 %)	30	(3,5 %)	6	(3,4 %)	87	(15,7 %)	4	(1,3 %)	83	(34,2 %)
Gastroenterologie	749	(10,2 %)	66	(7,7 %)	8	(4,5 %)	32	(5,8 %)	24	(7,7 %)	8	(3,3 %)
Andere	3994	(54,6 %)	111	(13,0 %)	35	(19,8 %)	260	(46,9 %)	192	(61,7 %)	68	(28,0 %)

± Standardabweichung, *min* Minimum, *max* Maximum, *N* Grundgesamtheit, *n* Teilmenge der Grundgesamtzeit, n Teilmenge der Fälle mit MMST

^a^Mini-Mental-Status-Test

**Figure 1:**
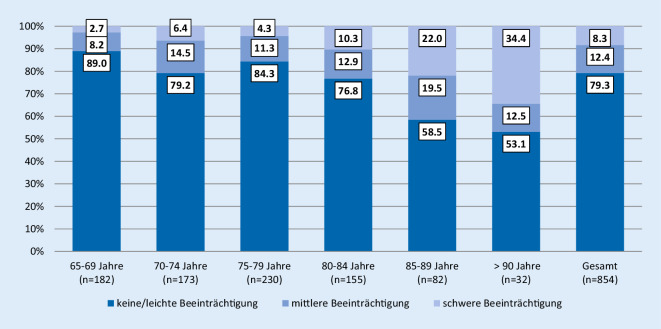

Insgesamt hatten 20,7 % der gescreenten Patienten eine leichte oder schwere kognitive Beeinträchtigung (*n* = 177). Die kognitive Beeinträchtigung steigt bei Patienten über 70 Jahren an (Abb. 1). Die Prävalenzrate für Demenz und Delir mittels F‑Diagnosen liegt für die Grundgesamtheit der über 65-jährigen bei 7,6 %.

### Bereitschaft zur Nutzung des Mini-Mental-Status-Tests

**Figure 2:**
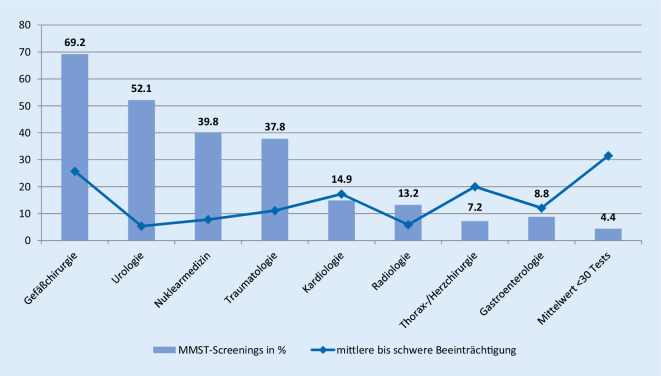

Insgesamt zeigen die Ergebnisse mit 11,7 % eine schwach ausgeprägte Bereitschaft, den MMST anzuwenden. Es gibt jedoch Unterschiede zwischen den Stationen resp. Organisationseinheiten (Abb. 2). In Einheiten mit einer niedrigeren Screeningrate sind die MMST-Scores, die für mittlere oder schwere kognitive Beeinträchtigungen sprechen, häufiger.

Die Prävalenz steigt mit dem Alter der Patienten an. Die Bereitschaft zu screenen steigt nicht mit dem Alter (Tab. 2). Die Verteilung der Screenings in der Grundgesamtheit und der gescreenten Fälle bleibt in den Altersgruppen gleich.

### Unterschiede zwischen den Prävalenzraten

Diagnosen von Demenz oder Delir wurden in 554 von 7311 Fällen (7,6 %) gefunden. Der χ^2^-Anpassungstest zeigt signifikant mehr durch den MMST gescreente kognitive Beeinträchtigungen, als mit medizinischen Diagnosen kodiert sind (χ^2^ (1) = 209,53, *p* < 0,001) (Tab. 3).

**Table 3:** 

Kognitiv beeinträchtigt (MMST^a^ < 24)	Beobachtete Häufigkeiten	Verteilung der F‑Diagnosen	Erwartete Häufigkeiten	Differenz	Differenz^2^	χ^2^
	B	*p*	E = n*p	B‑E	(B-E)^2^	(B-E)^2^/E
*Ja*	177	7,6	64,904	112,096	12.565,5132	193,601522
*Nein*	677	92,4	789,096	−112,096	12.565,5132	15,9239348
*Summe*	854	100	854	0	–	209,525457

^a^Mini-Mental-Status-Test, (*n* = 854; *p* < 0,001; 5 % Signifikanzniveau)

Durch die Anwendung des MMST von Pflegenden wurde ein größerer Anteil an kognitiv beeinträchtigten Patienten erkannt, als mit ICD-Codes kodiert wurden. Von allen Fällen mit MMST-Score hatten 67 auch eine Demenz- oder Delirdiagnose (Tab. 4). Von diesen 67 Fällen mit MMST-Screening und F‑Diagnose waren die Screeningresultate in 49 Fällen auffällig (73 %). Der MMST-Score führte zwar in mehr als 25 % der Fälle mit Demenz- oder Delirdiagnose zu keinem Argument für eine Kodierung einer kognitiven Beeinträchtigung. Der Zusammenhang zwischen einem MMST <24 und einer F‑Diagnose ist mit einem ɸ = 0,377 und einem ɸ_max_ = 0,57 dennoch deutlich gegeben und höchst signifikant (χ^2^ = 121,54, *p* = 0,001, α = 0,05) (Tab. 4).

**Table 4:** 

		MMST^a^ < 24		Summe
		Ja	Nein	
*F‑Diagnose*	*Ja*	49	18	67
	*Nein*	128	659	787
*Summe*		177	677	854

^a^Mini-Mental-Status-Test (ɸ = 0,377, ɸ_max_ = 0,571, χ^2^ = 121,54, *p* < 0,001)

## Diskussion

Die Prävalenzrate von 20,7 % kognitiv beeinträchtigten Patienten in der Gruppe der über 65-jährigen Krankenhauspatienten zeigt, dass mit der vorliegenden Untersuchung ein viel höherer Anteil von Patienten mit Symptomen einer Demenz oder eines Delirs in dieser Altersgruppe aufgezeigt werden konnte, als mit der Analyse von ICD-Codes möglich ist [23]. Die Resultate zeigen aber auch, dass zum Zeitpunkt des Screenings unauffällige Patienten (25 %) bei Entlassung eine Demenz- oder Delirdiagnose kodiert bekamen. Die Perspektiven auf den Patienten und seinen Versorgungsbedarf sind demnach je nach Zeitpunkt des Krankenhausaufenthaltes nicht immer kongruent. Diese Tatsache zeigt auch, dass für ein gemeinsames Verständnis eines Versorgungsprozesses ein Screening allein nicht ausreichend ist, sondern dass es auch Kommunikationsformate geben muss, sich über die betroffenen Fälle austauschen und eine gemeinsame Zielplanung erreichen zu können.

Die Untersuchung unterstreicht mit ihren Resultaten die Schlussfolgerung anderer Studien [23], dass die kognitiv beeinträchtigten Patienten häufig übersehen werden. Wie in dieser Studie zeigen die vorliegenden Ergebnisse eine ähnlich niedrige Prävalenz von Demenzdiagnosen (4,3 %). Allerdings muss hier berücksichtigt werden, dass die F‑Diagnosen häufig nicht kodiert werden, wenn sie für die Abrechnung irrelevant sind oder aufgrund fehlender Dokumentation in der Patientenakte nicht schlüssig zu begründen sind [22]. Die gescreenten kognitiven Beeinträchtigungen können auch durch Schmerzen, Angstzustände, medikamentöse Einwirkungen oder andere Gesundheitsstörungen bedingt sein, die nicht durch die ausgewählten F‑Diagnosen repräsentiert sind. Zudem muss bei der Interpretation der Ergebnisse berücksichtigt werden, dass die Patientenakten keine Informationen zum Bildungsstand der Patienten enthalten und eine Verzerrung des MMST-Scores durch Unter- oder Überschätzung möglich ist. Im Vergleich zu allen relevanten F‑Diagnosen bleiben die Unterschiede dennoch signifikant.

Die Ergebnisse zeigen je nach Altersgruppe Prävalenzen von mittleren und schweren kognitiven Beeinträchtigungen zwischen 10,9 und 46,9 % und bestätigen damit Ergebnisse anderer internationaler Arbeiten [17]. Wie bereits bekannt, steigt die Prävalenz mit dem Alter der Patienten an. Die Bereitschaft der Pflegenden, ein objektives Screeninginstrument zu nutzen, steigt jedoch nicht mit dem Alter der Patienten an. Mit nur 11,7 % zeigen die Ergebnisse eine schwache Bereitschaft, den MMST als Routineinstrument im Rahmen des Pflegeprozesses zu nutzen. Unterschiede gibt es zwischen den einzelnen Organisationseinheiten (7,2–69,2 %). Die Bereitschaft ist demnach womöglich von der Sensibilisierung und Einstellung der Pflegenden gegenüber Patienten mit kognitiven Beeinträchtigungen und von der Rolle der Multiplikatoren, Pflegeexperten und Vorgesetzten abhängig. Beispielsweise gibt es kein Screening von neurologischen Einheiten, obwohl das Bewusstsein für Patienten mit kognitiven Beeinträchtigungen in der neurologischen Pflege viel höher sein sollte als beispielsweise in der chirurgischen Pflege. Vielleicht ist das Erfahrungswissen der Pflegenden in diesen Bereichen der Grund, ein Screeninginstrument abzulehnen, da sie auf ihre eigenen Fähigkeiten zur klinischen Beurteilung vertrauen. Screenings werden möglicherweise erst als zweiter Schritt nach der klinischen Beurteilung gesehen. Eine andere Erklärung ist, dass möglicherweise viele Patienten den MMST aufgrund von Beeinträchtigungen beim Schreiben, beim Lesen oder beim Sprechen nicht ausfüllen können. Zwar wurden 45 unvollständige Tests ausgeschlossen, es ist jedoch auch möglich, dass noch mehr Patienten aufgrund ihrer Funktionsstörungen gar nicht erst eingeschätzt werden konnten. Daher kann die Bereitschaft, eine angemessenere Einschätzung mit dem MMST vorzunehmen, höher sein, als die Ergebnisse es vermuten lassen. Pflegende benötigen für eine Ersteinschätzung ein Instrument, welches unabhängig von motorischen Fähigkeiten des Patienten durchführbar, aber dennoch möglichst objektiv ist.

## Limitationen

Es gibt potenzielle Probleme bei der Verwendung von Routinedaten für eine Sekundärdatenanalyse. Es konnte nicht kontrolliert werden, dass alle Organisationseinheiten den gleichen Schulungsstand in der Durchführung des MMST haben. Darüber hinaus ist es möglich, dass Pflegende Patienten erst mit dem MMST untersuchen, wenn sie aufgrund ihres klinischen Fachwissens bereits eine kognitive Beeinträchtigung vermuten. Diese mögliche Verzerrung sollte bei der Verallgemeinerung der Ergebnisse dieser Studie berücksichtigt werden. Für Deutschland sind keine Erhebungen bekannt, die die Bereitschaft der Pflege, den MMST anzuwenden, untersucht haben. Aufgrund der Erfahrungen mit der Einführung anderer Screenings und Assessmentinstrumente ist es vermutlich auch der Faktor Zeit, der Pflegende davon abhält, den MMST anzuwenden resp. zu einer Rationierung von Pflegetätigkeiten im Kontext der Pflegeplanung und Dokumentation führt [24].

## Schlussfolgerungen

Kognitive Beeinträchtigungen sind bei älteren Krankenhauspatienten häufig, und Pflegekräfte benötigen ein geeignetes Einschätzungsinstrument, um betroffene Patienten frühzeitig zu identifizieren. Dass ein Fünftel der gescreenten Patienten über 65 Jahre kognitive Auffälligkeiten zeigt, bestätigt die Notwendigkeit, ein Routinescreening einzuführen, um den Versorgungsprozess passgenau planen zu können. Aufgrund der Unterschiede zwischen den Organisationseinheiten kann geschlussfolgert werden, dass das Personal etwa durch Pflegexperten weiterhin für diesen Themenbereich sensibilisiert und durch Demenzbegleiter unterstützt werden muss [19]. Der Zusammenhang von auffälligen MMST-Scores und den F‑Diagnosen ist zwar deutlich. Letztlich führt die niedrige Screeningrate aber zu dem Gedanken, ein anderes Instrument für den akut-stationären Bereich zu verwenden, mithilfe dessen sich kognitive Beeinträchtigungen leicht und schnell auch bei Patienten mit motorischen Funktionseinbußen erkennen lassen, z. B. den Six-Item Screener (SIS) [5, 11]. Durch den Anstieg auffälliger Screenings in den Altersgruppen über 69 Jahre erscheint es zudem effektiver, das routinemäßige Screening erst ab einem Alter ab 70 Jahren durchzuführen.

Es gibt einige Unterschiede zwischen den Ergebnissen des MMST-Scores und der Anzahl der medizinischen F‑Diagnosen in den Patientenakten. Die Ergebnisse unterstreichen die Notwendigkeit, das gesamte medizinische Personal, einschließlich der Ärzte in der Akutversorgung, für stationäre Patienten mit kognitiven Beeinträchtigungen zu sensibilisieren. Die Behandlung von kognitiven Beeinträchtigungen wie Delir liegt in der Verantwortung des gesamten Teams [8]. Für eine patientenzentrierte Versorgung und einen gemeinsamen Versorgungsplan mit gemeinsam vereinbarten Zielen müssen sich Verfahren für Screening, Diagnose und Kodierung von kognitiven Beeinträchtigungen in den Krankenhäusern entwickeln [19]. Hierbei sollten im weiteren Prozess Instrumente zum Einsatz kommen, die helfen, Delir und Demenz zu unterscheiden [12].

## Fazit für die Praxis

- Krankenhäuser sollten Verfahren entwickeln, um stationäre Patienten über 70 Jahre mit kognitiven Beeinträchtigungen frühzeitig zu erkennen.
- Der MMST wird als Routineinstrument in der Anamnese im Rahmen des Pflegeprozesses von Pflegenden zu wenig genutzt. In zukünftigen Projekten sollten die Bereitschaft und Praktikabilität der Anwendung kürzerer Instrumente, wie des Six-Item Screener (SIS) als initiales Screening untersucht werden.
- Die Anwendung von Screeninginstrumenten ist nur der erste Schritt für eine passgenaue Versorgungsplanung. Am Behandlungsprozess Beteiligte müssen für die Versorgungserfordernisse und Bedürfnisse der betroffenen Menschen sensibilisiert werden und begleitende Maßnahmen wie Demenzbegleiter, multiprofessionelle Kognitionsteams oder entsprechende Hilfsmittel zur Unterstützung haben.
